# The New York Institute of Stomatology

**Published:** 1904-12

**Authors:** 

**Affiliations:** The New York Institute of Stomatology


### Reports of Society Meetings.

### THE NEW YORK INSTITUTE OF STOMATOLOGY.

A MEETING of the Institute was held at the Chelsea, No. 222 West Twenty-third Street, New York, on Friday evening, June 3, 1904, the President, Dr. A. H. Brockway, in the chair.

The minutes of the last meeting were read and approved.

### COMMUNICATIONS ON THEORY AND PRACTICE.

Dr. C. O. Kimball presented a set of Dr. D. D. Smith's scalers upon which he had made some slight alterations that in his hands he had found to improve their utility. He had found them excellent for cleansing teeth.

*Dr. J. Morgan Howe.*—I have for some time wanted to express my appreciation and thanks to our fellow-member, Dr. Strang, for introducing the method of mixing amalgam and cement which he has recommended. I have found such mixture of the two materials to be so valuable that I think those who have not tried it would do well to do so. The use of a rather slow-setting amalgam and a quick-setting cement works best, in my estimation. The filling being mixed and introduced, in five or six minutes the filling can be shaped and margins made flush; then lightly rubbing the

---

surface with a burnisher will cause the particles of amalgam to coalesce, giving practically an amalgam surface. This method will prevent the filling from wearing rough, at least in a great degree.

*Dr. S. E. Davenport.*—Will Dr. Howe kindly tell us for what class of cases he thinks this combination is best adapted?

*Dr. Howe.*—The filling is adapted for those cases where adhesion to the walls of the cavity is desirable, the cement giving the filling the adhesive qualities, while the amalgam protects the cement and gives durability. Cavities that run under the margin of the gum, filled with it, have made a good record in mouths where cement alone would quickly waste away and gutta-percha had proved to be practically worthless.

All conditions unfavorable to the retention of the filling because of the shape of the cavity or the weakness of the walls are indications for this mixture of amalgam and cement.

*Dr. F. Milton Smith.*—I would be willing to part with almost anything else in the way of a filling-material rather than with this combination. I believe that almost any one will make a failure in the use of this filling-material if he does not use the utmost care, following the little details of preparation according to Dr. Strang's directions. I had in my office to-day a patient for whom I inserted one of my first fillings of this kind, a posterior cavity in an upper first molar. It was something over five years ago, and the tooth is practically perfectly preserved. I am sure that in that time it would have been filled four or five times with either cement or amalgam. I have had almost uniform success, although I have had cases where the combination has worn away quite rapidly. This, however, I have ascribed to some fault of my own in the mixing.

*The President.*—I have used this combination in many cases with great satisfaction. Dr. Strang does not claim that he originated this method, but gives the credit, I believe, to Dr. Tillotson. Our Dr. C. B. Parker, I remember, read a paper describing it several years ago.

*Dr. Smith.*—Regarding the proper proportions for this mixture, the amount of cement powder is one-half as much as the amount of filings in bulk. The filings are amalgamated first and placed in a mortar with the oxide of zinc and the two ground to almost an impalpable powder. It is then placed on the slab and mixed with the liquid of the cement, the mass then being kneaded with the fingers for a little while. A matrix should always be used for approximal cavities, and the filling inserted with pressure. Then a thorough burnishing over the surface makes a filling that will last.

*Dr. C. O. Kimball.*—Might I modify Dr. Smith's excellent method? I too am very much indebted to Dr. Strang, and have used this method continually. I do not altogether like the "rule-of-thumb" method followed by Dr. Smith. I have the powders taken out separately and placed in two heaps. I prefer a rather slow-setting cement, and find the Dirigo very satisfactory for this purpose. The amalgam is mixed dry, rubbing it up in a mortar, not touching it with the hands at all. The powder is then placed in the mortar and thoroughly incorporated with the amalgam. It is then placed on the slab and mixed with the phosphoric acid, then not rubbed in the hand but with the spatula until the right stiffness is obtained, which can be judged by the feeling of the spatula. It is not rolled in the hand, but taken up by the spatula and put directly into the cavity, where it is thoroughly and rapidly moulded into the required contour. I think it is well, as far as possible, to avoid all contact with the hand, and above all the tooth must be kept perfectly and absolutely dry from the beginning to the end of the operation. A little fresh amalgam may be burnished over the surface after the proper shape has been given to the filling. If carefully done it will make a filling that will stand in a wonderful way.

*Dr. Fossume.*—I wish to present for your inspection a set of instruments made of ivory for burnishing inlay matrices. These instruments will not iron or stretch the matrix, and thereby overcome, to a great extent, the rigidity and pulling away of the metal.

I also pass around some aseptic dental-bracket covers that Johnson & Johnson are putting on the market. They are made in three sizes, for the Allen, Holms, and Harvard tables, and are put up in boxes containing from one hundred to five hundred covers. The idea is to get an aseptic cover at a small cost, so that they can be changed after each patient. These covers will be supplied, I believe, at seventy-five cents a hundred.

*Dr. J. Morgan Howe.*—Mr. President and gentlemen, the subject of malignant growths in the mouth has interested me for a number of years, not because I know anything about it, but because a good many cases have come under my observation. I have been willing to speak upon the subject this evening in order to urge upon dentists the desirability of their giving attention to the subject, that they may early recognize any suspicious indications. I have seen several cases where the patient was passed from dentist to physician and back to the dentist again, the physician supposing the trouble to originate from the root of a tooth and the dentist not being certain whether it was a tooth or not, the patient being encouraged to await developments, action being postponed until—in one case—fatal results followed. I got the patient into the hands of a surgeon,—the late Dr. Garretson,—and he was operated upon the very same day that I saw him first, but the tumor recurred. I would not urge upon dentists the study of malignant growths with any other object than to have their suspicions aroused when they should be. I have in mind a patient who, when I suggested that a section for examination be made of a tumor, stated that this had already been done and the tumor pronounced benign, the patient giving the name of a dentist whom I knew to have given some attention to microscopy and histology. My examination of the case was some months later. I would not advise dentists to set themselves up as pathologists, but to make such a study of malignant growths as to be able to give patients good advice. It not infrequently happens that patients are needlessly alarmed about some little thing that need not be suspected of malignancy, and the dentist is fortunate who can dissipate such fears.

We are very fortunate in having with us to-night a gentleman who can tell you a great deal about this subject. I will say, in this connection, that I have been very much interested in reading Dr. Dawbarn's book, for which he received the Gross prize. The book contains a description of diseased growths in a good many parts of the body and not a few about the mouth. It is one of the books that is well worth the attention of dentists.

Of course it is understood that in speaking of this subject I merely call attention to it from a dentist's point of view. We expect the information from the gentlemen who are to follow.

(For Dr. Dawbarn's paper on "Malignant Growths in the Mouth," see page 911; for Dr. Franklin's paper on "Radiotherapy in some Malignant Conditions of the Mouth," see page 905.)

*Dr. Dawbarn.*—In the main I have nothing to differ from Dr. Franklin's very interesting paper, except in one respect. If I understood him correctly, in cancer of the tongue he would advise using the X-ray instead of the knife. I have known of two instances of cancer in this locality where the patient lost his life in just this manner. There is no exception to the rule that the proper thing to do is to use the knife at once. The doctor refers to the great danger of the application of the X-ray to deep-seated conditions. In this connection the value of the X-ray in locating gall-stones is of great value. Also in fractures of the thigh-bone or in fracture of any of the bones it aids materially in properly reducing the fracture.

*Dr. Davenport.*—I hope, Mr. President, you will extend our cordial thanks to these gentlemen for what they have done for us this evening. Dr. Dawbarn being one of us, perhaps we may take his efforts in our behalf more as a matter of course, though we should not be less appreciative of his many services to this society. From what we have learned of Dr. Franklin to-night I am sure we all hope he will be a frequent visitor.

*Dr. Kimball.*—In seconding Dr. Davenport's suggestion, I wish to state that while we cannot very well discuss this subject, the society certainly owes a debt to these gentlemen for giving us this information so clearly and forcibly.

*Dr. Franklin.*—Regarding the point upon which Dr. Dawbarn differs from me, I really do not believe that he differs from me, or that I differ from him, so much as he thinks. While it has been my custom to X-ray cases of the tongue first, at the very first sign of lack of response or of further involvement I turn the case over to the surgeon. A reason why the ray has been used so much in cases that should have been operated upon is to be found in the fact that there exists in the hearts of most women an extreme dread of surgical interference. Cases have come to me in which I have actually begged the patient to place herself in the hands of a surgeon, and insisted that every day the growth was X-rayed the danger was increasing. In many cases they have been obdurate. In many cases they have gone to others who have held out more hope. I know of two cases that have died because they would not allow themselves to be operated upon.

*The President.*—Before entertaining a motion to adjourn, I wish to tender to Dr. Dawbarn and Dr. Franklin the thanks of the society for their instructive and interesting papers.

Adjourned.

Fred. L. Bogue, M.D., D.D.S.,
*Editor The New York Institute of Stomatology.*

